# Is histological grade a useful parameter in muscle-invasive urothelial bladder cancer? Results from a multicenter study on the impact of different grading systems on disease-free survival after upfront radical cystectomy

**DOI:** 10.1007/s00345-025-06089-z

**Published:** 2025-11-20

**Authors:** Emily Rinderknecht, Francesco Claps, Peter J. Bostrom, Shahrokh F. Shariat, Yann Neuzillet, Alexandre R. Zlotta, Carlo Trombetta, Markus Eckstein, Renee A. G. Lijnen, Laura S. Mertens, Rossana Bussani, Maximilian Burger, Geert J. L. H. van Leenders, Joost L. Boormans, Bernd Wullich, Arndt Hartmann, Nicola Pavan, Damien Pouessel, Theo H. van der Kwast, Yves Allory, Tahlita C. M. Zuiverloon, Yair Lotan, Bas W. G. van Rhijn, Roman Mayr

**Affiliations:** 1 Department of Urology, Caritas St. Josef Medical Center, University of Regensburg, Landshuter Straße 65, 93053 Regensburg, Germany; 2 Department of Surgical Oncology (Urology), Netherlands Cancer Institute – Antoni van Leeuwenhoek Hospital, Amsterdam, The Netherlands; 3 Department of Medicine, Surgery and Health Sciences, Urological Clinic, University of Trieste, Trieste, Italy; 4 Department of Surgery (Urology) and Surgical Oncology, Princess Margaret Cancer Center, University Health Network, University of Toronto, Toronto, ON Canada; 5 Department of Urology, Turku University Hospital and University of Turku, Turku, Finland; 6 Department of Urology, University of Texas Southwestern Medical center, Dallas, TX USA; 7 Department of Urology, Comprehensive Cancer Center, Medical University of Vienna, Vienna, Austria; 8 Department of Urology, Weill Cornell Medical College, New York, NY USA; 9 Department of Urology, Second Faculty of Medicine, Charles University, Prague, Czech Republic; 10 Institute for Urology and Reproductive Health, I.M. Sechenov First Moscow State Medical University, Moscow, Russia; 11 Molecular Oncology team, Institut Curie, CNRS, UMR144, PSL Research University, Paris, F-75005 France; 12 Department of Urology, Hôpital Foch, UVSQ-Paris-Saclay University, Suresnes, 92150 France; 13 Institute of Pathology, University Hospital Erlangen, Friedrich-Alexander-Universität Erlangen-Nürnberg, Erlangen, Germany; 14 Department of Pathology, University of Trieste, Trieste, Italy; 15 Department of Pathology, Erasmus MC Cancer Institute, University Medical Center, Rotterdam, The Netherlands; 16 Department of Urology, Erasmus MC Cancer Institute, University Medical Center, Rotterdam, The Netherlands; 17 Department of Urology & Pediatric Urology, University Hospital Erlangen, Friedrich-Alexander-Universität Erlangen- Nürnberg, Erlangen, Germany; 18 Department of Precision Medicine in Medical, Surgical and Critical Care, Urological Clinic, University of Palermo, Palermo, Italy; 19 Department of Medical Oncology, Oncopole Claudius Regaud, Toulouse University Cancer Center (IUCT) Oncopole, Toulouse, F-31000 France; 20 Toronto and Laboratory Medicine and Pathobiology, Princess Margaret Cancer Center, University of Toronto, Toronto, Canada; 21 Department of Pathology, Institut Curie, Paris, F-75005 France; 22 Department of Urology, University of Augsburg, Stenglinstrasse 2, 86156 Augsburg, Germany

**Keywords:** Urothelial neoplasm, Urothelial carcinoma, Histopathological grade, WHO1973 classification, WHO2004 classification, Radical cystectomy

## Abstract

**Purpose:**

The prognostic value of histopathological grade in muscle-invasive urothelial carcinoma (MIBC) to predict disease-specific survival (DSS) is understudied. While grading systems like WHO1973 and WHO2004 are established in non-muscle-invasive bladder cancer (NMIBC), their relevance in MIBC remains controversial. This study assessed the prognostic impact of histopathological grade on DSS in a multicenter cohort.

**Methods:**

We included 1,123 cN0M0 MIBC patients treated with upfront radical cystectomy (1987–2020) at nine centers. Tumors were graded using WHO1973 (G1 + G2 combined as G1/2 due to low numbers vs. G3), WHO2004 (low-grade [LG] vs. high-grade [HG]), and a hybrid three-tier system. Slides were locally reviewed by uro-pathologists. DSS was analyzed using Kaplan-Meier and Cox models, adjusting for age, stage, lympho-vascular invasion, surgical margins, lymph-node status, adjuvant chemotherapy, treatment center, and era of cystectomy.

**Results:**

Among all cases, 74 (6.6%) were G1/2 and 1,049 (93.4%) G3; 27 (2.4%) were LG and 1,096 (97.6%) HG. Median follow-up was 5.3 years (IQR 2.9–8.5). Univariable analyses showed significantly better DSS for LG and G1/2 tumors across grading systems. However, multivariable models showed no independent association between grade and DSS.

**Conclusion:**

Although LG and G1/2 MIBC tumors demonstrated superior DSS in univariable analyses, the lack of independent prognostic significance in multivariable models questions the relevance of histopathological grade in MIBC. Further studies should explore the clinical utility of grade, define new grading schemes including features of epithelial-mesenchymal transition or tumor microenvironment, and explore alternative prognostic (bio)markers.

**Supplementary Information:**

The online version contains supplementary material available at 10.1007/s00345-025-06089-z.

## Introduction

The role of histopathological grade in muscle-invasive urothelial carcinoma (MIBC) is not clear. While grading systems such as the 1973 and 2004 World Health Organization (WHO) classifications have been used to predict outcomes in non-muscle-invasive bladder cancer (NMIBC) [1], their utility in MIBC remains controversial and understudied. The current European Association of Urology (EAU) guidelines on muscle-invasive and metastatic bladder cancer even state that grade does not provide any prognostic information in MIBC [2]. However, they also recommend that pathologists specify the tumor grade during the primary assessment of presumed MIBC [2].

For a long time, the WHO1973 classification was the only system used, in which tumors were classified histopathologically into grades 1, 2, or 3 (G1, G2 or G3). The 2004 classification was introduced to add detailed histological criteria and to address the pathologist’s preference for G2 (WHO1973). This resulted in the binary system of low grade (LG) and high grade (HG) cancer and added a separate, non-cancer entity, i.e. Papillary Urothelial Neoplasm of Low Malignant Potential (PUNLMP) [3–6]. While the WHO2004 classification is most commonly used in the United States to grade NMIBC [7], European guidelines recommend the parallel use of both grading systems (WHO1973 and 2004) [8]. The current 2022 edition of the WHO classification recommends the continued use of the WHO2004 grading system for both non-muscle-invasive and muscle-invasive bladder cancer [9]. Recently, a three-tier hybrid system has also been proposed, which separates WHO2004 HG-lesions into HG/G2 and HG/G3 while maintaining LG as a separate category [6, 10–12].

Although both the WHO1973 and the WHO2004 classifications can provide significant prognostic information in NMIBC, especially for prediction of progression [6, 13], research on grade in MIBC remains very limited [2]. This further underscores the need to reassess histopathological grade in MIBC. The aim of our study was to explore whether histopathological grade in MIBC is useful by analyzing the impact of grade on disease specific survival (DSS) in a multi-center cohort of upfront radical cystectomy (RC) patients whose pathology was reviewed.

## Patients and methods

### Patients, treatment, and follow-up

We included a total of 1,123 patients from nine uro-oncology centers in Europe and North America (Table 1). All patients had MIBC (cN0M0) and were treated with upfront RC between 1987 and 2020. Grading was available according to both WHO1973 and WHO2004 classifications, based on local pathology review of the cystectomy specimens. Pure non-urothelial subtypes were excluded. The pathology review was conducted prior to the 2022 update of the WHO2004 classification, which - at the time - allowed histological subtypes to be graded as LG, a classification no longer permitted under the current version of WHO2004 criteria, where all subtypes are classified as HG.

Follow-up regimes were determined by the treating physician. Adjuvant systemic chemotherapy was permitted and was given at the discretion of the treating physicians in accordance with the then-current clinical guidelines and institutional practice. Data on baseline characteristics (age, gender), pathological outcome (grade, positive margins, nodal status, lympho-vascular invasion [LVI], concomitant carcinoma in situ) and survival outcome were collected. DSS was defined as the time from radical cystectomy to death from bladder cancer, patients alive at last follow-up or who died from other causes were censored. Ethical approval was granted.

### Statistical analysis

Absolute and relative frequencies were reported for categorical variables. For metric variables, median values with interquartile range (IQR) were reported. Comparative analyses between groups were performed using the Chi-squared test, as appropriate.

DSS was shown using Kaplan-Meier curves. For all analyses, G1 and G2 of the WHO1973 classification were merged due to the anticipated low incidence of G1-cases. Kaplan-Meier estimates were compared using the log-rank test. Regarding the three-tier Hybrid classification system, a Log-Rank test for trend was conducted in univariable analysis. Cox proportional hazard models were used to compare the prognostic value for the different classification systems for grade, adjusting for age, stage, LVI, margins, nodes, adjuvant chemotherapy, treatment center, and era of cystectomy. These variables were selected based on clinical relevance and on significance in univariable analyses (*p* < 0.05). There were no missing data for any of the variables included in the analyses. Multi-collinearity among variables was assessed using variance inflation factors (VIF), for categorical predictors with more than two levels, generalized VIF was calculated. VIF values < 5 were considered acceptable. In addition to the analysis of the overall cohort, a sensitivity analysis limited to patients with pure urothelial carcinoma was conducted to evaluate the robustness of the multivariable Cox regression results. Patients without events were censored at their last date of follow-up. Median follow-up was calculated using only the data of patients without event.

All p-values were two-tailed and considered statistically significant at a threshold of < 0.05. Statistical analyses were performed with IBM SPSS Statistics for Windows, version 29.0 (IBM Corp., Armonk, NY, USA) and R version 4.4.2 (R Foundation for Statistical Computing, Vienna, Austria) accessed via R-Studio (Posit Software, Boston, MA, USA). Graphical data representation was conducted using GraphPad Prism, version 10 (GraphPad Software; San Diego, CA, USA).

## Results

### Patient characteristics

A total of 1,123 cases were included in the analysis. Patient characteristics are presented in Table 1 (entire cohort) and Tables 2, 3 and 4 (stratified according to grading systems). Of the patients, 867 (77.2%) were male. The median age was 67.5 years (IQR: 59.0–74.8.0.8 year). Of the patients, 311 (27.2%) were pT2, referred to as organ-confined disease, while 577 (51.4%) and 235 (20.9%) had locally advanced disease, with stages pT3 and pT4, respectively. Of pT2 tumors, 38 (12.2%) were G1/2, and 273 (87.8%) G3, while 15 (4.8%) were LG and 296 (95.2%) HG. In pT3/4 tumors, 36 (4.4%) were G1/2, and 776 (95.6%) G3, while 12 (1.5%) were LG and 800 (98.5%) HG. There were histological subtypes of urothelial carcinoma as opposed to pure urothelial carcinoma in 289(25.7%) of patients. LVI was absent (LVI-) in 549 (48.9%) patients and present (LVI+) in 574 (51.1%) patients. In LVI- tumors, 44 (8.0%) were G1/2, and 505 (92.0%) G3, while 18 (3.3%) were LG and 531 (96.7%) HG. In LVI + tumors, 30 (5.2%) were G1/2, and 554 (94.8%) G3, while 9 (1.6%) were LG and 565 (98.4%) HG. There was no lymph-node involvement (pN0) in 669 (59.6%) of the patients, while 545 (40.4%) had positive lymph nodes (pN+). In pN0 tumors, 55 (8.2%) were G1/2, and 614 (91.8%) G3, while 22 (3.3) were LG and 647 (96.7%) HG. In pN + tumors, 19 (4.2%) were G1/2, and 435 (95.8%) G3, while 5 (1.1%) were LG and 449 (98.9%) HG. DSS was 51.1% at a median follow-up of 5.31 years (IQR: 2.90–8.54 year).

**Table 1:** Clinicopathological characteristics of the study cohort (*n* = 1,123)

	Cohort *n* = 1,123
City	
Amsterdam (%)	174(15.5)
Paris (%)	162(14.4)
Regensburg (%)	156(13.9)
Rotterdam (%)	147(13.1)
Trieste (%)	145(12.9)
Dallas (%)^a^	121(10.8)
Erlangen (%)	98(8.7)
Toronto (%)	81(7.2)
Turku (%)^a^	39(3.5)
Gender (male, %)	867(77.2)
Median age (IQR, yr)	67.5(59.0–74.8.0.8)
Year of RC	
1987–2000 (%)	255(22.7)
2001–2010 (%)	678(60.4)
2011–2020 (%)	190(16.9)
Stage	
pT2 (%)	311(27.7)
pT3 (%)	577(51.4)
pT4 (%)	235(20.9)
Grade WHO1973	
G1 (%)	1(0.1)
G2 (%)	73(6.5)
G3 (%)	1049(93.4)
Grade WHO2004	
LG (%)	27(2.4)
HG (%)	1,096(97.6)
Hybrid Grade	
LG (%)	27 (2.4)
HG/G2 (%)	47 (4.2)
HG/G3 (%)	1049 (93.4)
Histological Subtype (%)	289(25.7)
CIS at RC (CIS, %)	385(34.3)
LVI at RC (LVI, %)	574(51.1)
Margins (positive, %)	114(10.2)
Nodes (positive, %)	454(40.4)
Adjuvant chemotherapy (%)	429(38.2)
DSS (dead of disease, %)	574(51.1)
Time to follow-up DSS (IQR, yr)^b^	5.31 (2.90–8.54)

Values are presented as number (percentage) unless otherwise specified.

Median age and follow-up time are shown with IQR.

^a^ Slides of the Dallas and Turku cohorts were reviewed by TvdK (Toronto).

^b^ median follow-up time was calculated using the reverse Kaplan-Meier method, accounting for censored patients as events.

CIS = carcinoma in situ; DSS = disease-specific survival; HG = high grade; IQR = interquartile range; LG = low grade; LVI = lympho-vascular invasion; RC = radical cystectomy; UC = urothelial carcinoma; yr = years.

**Table 2:** Clinicopathological characteristics stratified by Grade, WHO1973, dichotomized into G1/2 and G3. Values are presented as number (percentage)^a^ unless otherwise specified. The distribution of categorial variables was compared between G1/2 and G3 tumors

	Entire Cohort *n* = 1,123	G1/2 *n* = 74	G3 *n* = 1,049	*p*-value^b^
Median age (IQR, yr)	67.5(59.0–74.8.0.8)	66.7(58.0–76.0)	67.6(59.0–74.7.0.7)	
Stage				< 0.001
pT2 (%)	311(27.7)	38(51.4)	273(26.0)	
pT3 (%)	577(51.4)	27(36.5)	550(52.4)	
pT4 (%)	235(20.9)	9(12.2)	226(21.5)	
Histological Subtype (%)	289(25.7)	11(14.9)	278(26.5)	0.027
LVI at RC (LVI, %)	574(51.1)	30(40.5)	544(51.9)	0.06
Nodes (positive, %)	454(40.4)	19(25.7)	435(41.5)	0.007
Margins (positive, %)	114(10.2)	2(2.7)	112(10.7)	0.028
Adj. chemotherapy (%)	429(38.2)	25(33.8)	404(38.5)	0.418

^a^ Note: Groups are strongly imbalanced towards G3; percentages in smaller groups (G1/2) should be interpreted with caution.

^b^ Group comparisons were performed using the Chi-squared test.

Adj. = adjuvant; IQR = interquartile range; LVI = lympho-vascular invasion; RC = radical cystectomy; yr = years.

**Table 3:** Clinicopathological characteristics stratified by Grade, WHO2004. Values are presented as number (percentage)^a^ unless otherwise specified. The distribution of categorial variables was compared between LG and HG tumors

	Entire Cohort *n* = 1,123	LG *n* = 27	HG *n* = 1,096	*p*-value^b^
Median age (IQR, yr)	67.5(59.0–74.8.0.8)	66.8(57.7–74.9)	67.6(59.0–74.8.0.8)	
Stage				0.005
pT2 (%)	311(27.7)	15(55.6)	296(27.0)	
pT3 (%)	577(51.4)	8(29.6)	569(51.9)	
pT4 (%)	235(20.9)	4(14.8)	231(21.2)	
Histological Subtype (%)	289(25.7)	2(7.4)^c^	287(26.2)	0.027
LVI at RC (LVI, %)	574(51.1)	9(33.3)	565(51.6)	0.016
Nodes (positive, %)	454(40.4)	5(18.5)	449(41.0)	0.019
Margins (positive, %)	114(10.2)	1(3.7)	113(10.3)	0.261
Adj. chemotherapy (%)	429(38.2)	6(22.2)	423(38.6)	0.084

^a^ Note: Groups are strongly imbalanced towards HG; percentages in smaller groups (LG) should be interpreted with caution.

^b^ Group comparisons were performed using the Chi-squared test.

^c^ Pathology review was conducted prior to 2022. Histological subtypes are now automatically classified as HG.

Adj. = adjuvant; HG = high grade; IQR = interquartile range; LG = low grade; LVI = lympho-vascular invasion; RC = radical cystectomy; yr = years.

**Table 4:** Clinicopathological characteristics stratified by Grade, hybrid grading scheme. Values are presented as number (percentage)^a^ unless otherwise specified. Median age is shown with IQR. The distribution of categorial variables was compared between LG, HG/G2 and HG/G3 tumors

	Entire Cohort *n* = 1,123	LG *n* = 27	HG/G2 *n* = 47	HG/G3 *n* = 1,049	*p*-value^b^
Median age (IQR, yr)	67.5(59.0–74.8.0.8)	66.8(57.7–74.9)	66.6(58.3–77.4)	67.6(59.0–74.7.0.7)	
Stage					< 0.001
pT2 (%)	311(27.7)	15(55.6)	23(48.9)	273(26.0)	
pT3 (%)	577(51.4)	8(29.6)	19(40.4)	550(52.4)	
pT4 (%)	235(20.9)	4(14.8)	5(10.6)	226(21.5)	
Histological Subtype (%)	289(25.7)	2(7.4)^c^	9(19.1)	278(26.5)	0.047
LVI at RC (LVI, %)	574(51.1)	9(33.3)	21(44.7)	544(51.9)	0.109
Nodes (positive, %)	454(40.4)	5(18.5)	14(29.8)	435(41.5)	0.018
Margins (positive, %)	114(10.2)	1(3.7)	1(2.1)	112(10.7)	0.088
Adj. chemotherapy (%)	429(38.2)	6(22.2)	19(40.4)	404(38.5)	0.216

^a^ Note: Groups are strongly imbalanced towards HG/G3; percentages in smaller groups (LG; HG/G2) should be interpreted with caution.

^b^ Group comparisons were performed using the Chi-squared test.

^c^ Pathology review was conducted prior to 2022. Histological subtypes are now automatically classified as HG.

Adj. = adjuvant; HG = high grade; IQR = interquartile range; LG = low grade; LVI = lympho-vascular invasion; RC = radical cystectomy; yr = years.

#### Disease-specific survival in univariable analysis.

**Fig. 1:**
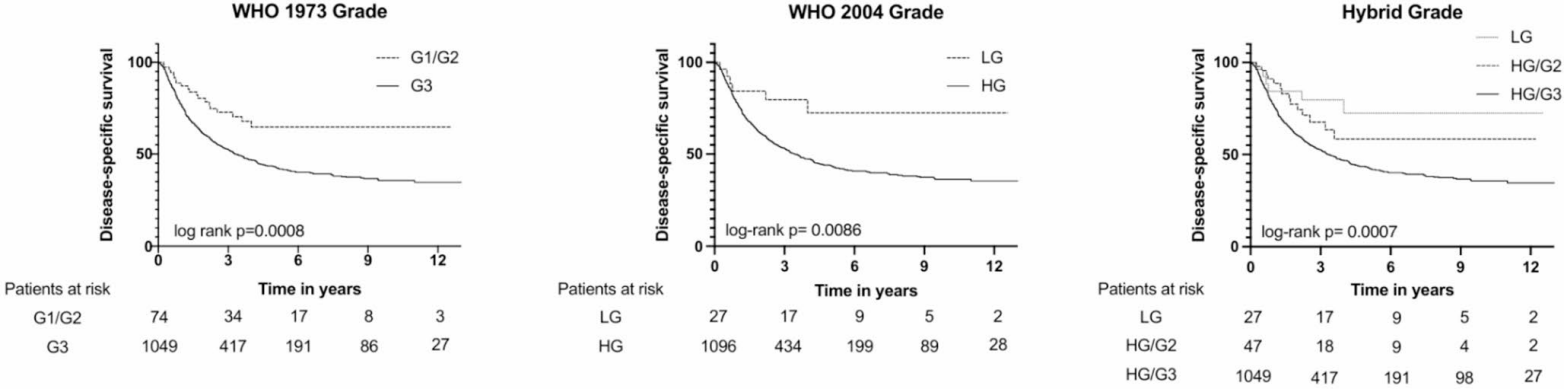
Kaplan-Meier curves illustrating disease-specific survival according to the three histopathological grading systems: WHO 1973, WHO 2004, and Hybrid classification. Differences between curves were assessed using the log-rank test. HG = high grade; LG = low grade

Figure1 shows the Kaplan-Meier curves for DSS in the MIBC cohort according to the WHO1973 and WHO2004 classification systems, as well as the Hybrid three-tier grading scheme. Patients with G3 according to WHO1973 had a significantly shorter DSS compared to those with G1/2 (median DSS of 3.3 years vs. not reached, *p* < 0.001). Patients with HG according to WHO2004 had a significantly shorter DSS compared to those with LG (median DSS of 3.42 years vs. not reached, *p* = 0.009). Regarding the Hybrid grading scheme, we found significant differences in DSS between the three groups HG/G3, HG/G2 and LG (median DSS of 3.3 years vs. not reached vs. not reached, p = < 0.001). Post-hoc pairwise comparisons were performed to examine the differences between individual groups. The results revealed no significant difference in DSS between LG and HG/G2 (*p* = 0.348), but significant differences were observed between LG and HG/G3 (*p* = 0.007), and between HG/G2 and HG/G3 (*p* = 0.03).

Among a subgroup with generally favorable pathological features, including the 186 patients with pT2, pN0, and LVI-, DSS proved significantly shorter for those with G3 tumors compared to those with G1/2 tumors (median DSS of 11 years vs. not reached, *p* = 0.037). Stratifying DSS according to the WHO2004 or Hybrid classifications in the same subgroup, no significant differences in DSS were observed (*p* = 0.299 for WHO2004, *p* = 0.079 for Hybrid).

#### Multivariable analysis

The base model included age, stage, LVI, margins, nodes, adjuvant chemotherapy, treatment center (modelled as frailty/random effect), and era of cystectomy. The different grading schemes were individually incorporated to create three additional multivariable models. Variance inflation factors indicated no problematic multi-collinearity among predictor variables (all VIF < 5, Supplementary Table S1).

The results regarding the entire cohort are presented in Table 5. The results regarding only patients with pure urothelial carcinoma are presented in Supplementary Table S2.

In the final multivariable model regarding dichotomized WHO1973 grading, G3 as opposed to G1/2 was not significantly associated with decreased DSS (HR = 1.48, 95%CI: 0.94–2.33, *p* = 0.093). WHO2004 grade was not significantly associated with DSS (HR = 1.90, 95%CI: 0.84–4.29, *p* = 0.122). For the Hybrid grading scheme, which included three categories (LG, HG/G2 and HG/G3), LG served as the reference-group. After adding the Hybrid-scheme variable to the model, neither HG/G2 (HR = 1.49, 95%CI: 0.57–3.91, *p* = 0.419) nor HG/G3 (HR = 1.92, 95%CI: 0.85–4.33, *p* = 0.116) were significantly associated with DSS. Furthermore, when HG/G3 was used as the reference group, comparing HG/G2 with HG/G3 showed no significant association with DSS (HR = 0.78, 95%CI: 0.45–1.34, *p* = 0.361).

**Table 5:** Multivariable Cox-Regression analysis of the impact of the WHO1973, WHO2004, and hybrid grading scheme, including the base model, on disease-specific survival (*n* = 1,123)

	base model		WHO 1973^a^		WHO 2004		Hybrid (Ref: LG)	
Variable	HR (95% CI)	*p*-value	HR (95% CI)	*p*-value	HR (95% CI)	*p*-value	HR (95% CI)	*p*-value
age (years)	1.01 (1.00–1.02.00.02)	0.063	1.01 (1.00–1.02.00.02)	0.059	1.01 (1.00–1.02.00.02)	0.055	1.01 (1.00–1.02.00.02)	0.056
stage (Ref: pT2)								
pT3	1.51 (1.20–1.89)	< 0.001	1.47 (1.17–1.84)	< 0.001	1.49 (1.19–1.86)	< 0.001	1.47 (1.17–1.84)	< 0.001
pT4	1.97 (1.51–2.57)	< 0.001	1.93 (1.48–2.51)	< 0.001	1.95 (1.50–2.55)	< 0.001	1.93 (1.48–2.52)	< 0.001
LVI	1.41 (1.17–1.71)	< 0.001	1.41 (1.16–1.70)	< 0.001	1.40 (1.16–1.69)	< 0.001	1.40 (1.16–1.69)	< 0.001
positive nodes	2.09 (1.72–2.55)	< 0.001	2.07 (1.69–2.52)	< 0.001	2.08 (1.70–2.54)	< 0.001	2.07 (1.69–2.52)	< 0.001
positive margins	1.39 (1.08–1.80)	0.011	1.39 (1.07–1.79)	0.012	1.39 (1.08–1.80)	0.011	1.39 (1.08–1.79)	0.012
adj. chemotherapy	0.89 (0.72–1.10)	0.274	0.89 (0.73–1.10)	0.292	0.89 (0.73–1.10)	0.290	0.90 (0.73–1.10)	0.295
era of CE (Ref: ≤ 2000)								
2001–2010	1.05 (0.83–1.33)	0.669	1.04 (0.82–1.32)	0.749	1.05 (0.83–1.34)	0.675	1.04 (0.82–1.32)	0.727
2011–2020	1.14 (0.80–1.63)	0.459	1.14 (0.80–1.62)	0.463	1.14 (0.80–1.63)	0.465	1.14 (0.80–1.62)	0.465
Treatment center^b^	-^c^		-^d^		-^e^		-^f^	
+ Grading	**-**	**-**	**1.48 (0.94–2.33)**	**0.093**	**1.90 (0.84–4.29)**	**0.122**	HG/G2 **1.49 (0.57–3.91)**	**0.419**
							HG/G3 **1.92 (0.85–4.33)**	**0.116**

^a^ dichotomized into G1/2 and G3. ^b^ treatment center was included as a frailty (random effect). ^c^variance of the term = 0.0225, p-value from likelihood-ratio test = < 0.001. ^d^variance of the term = 0.0204, p-value from likelihood-ratio test = < 0.001. ^e^variance of the term = 0.0246, p-value from likelihood-ratio test = < 0.001. ^f^variance of the term = 0.0220, p-value from likelihood-ratio test = < 0.001.

Adj. = adjuvant; CE = cystectomy; CI = confidence interval; HG = high grade; HR = hazard ratio; LG = low grade; LVI = Lympho-vascular invasion; Ref = reference category.

## Discussion

The present multicenter, real-world cohort-study investigated whether histopathologic grade is prognostically relevant in patients with MIBC. The first important result of the study is that LG and/or G1/G2 differentiated tumors do exist in MIBC, although they are rare. Notably, some tumors exhibited favorable histologic grade (LG or G1/G2) despite being associated with otherwise adverse pathological features, such as advanced tumor-stage, LVI, and nodal involvement. While such tumors were exceedingly rare, they do occur. Secondly, these LG or G1/G2 differentiated tumors exhibited significantly superior DSS across all three grading schemes in univariable analysis (Fig. 1). However, in the multivariable analyses adjusting for age, stage, LVI, lymph-node status, surgical margins, adjuvant chemotherapy, treatment center and era of radical cystectomy, none of the grading schemes significantly predicted DSS in MIBC, whereas established variables such as pT-stage, pN-stage, and LVI remained independently prognostic. The absence of an independent effect of grade in our cohort likely reflects the predominance of higher grade (G3 or HG) disease in MIBC, limiting the discriminative potential of histologic grading once tumor stage and other variables are accounted for. Within the Hybrid Grading system, the relatively higher proportion of G1/2 compared with LG cases - although both categories were uncommon - may have further reduced statistical power to detect a meaningful association. Yet, although some lower grade tumors exhibited otherwise adverse pathological features, higher grade tumors were generally enriched for adverse features such as higher pT-stage, nodal involvement, and LVI (Tables 4a-c). Consequently, the significant differences observed in univariable analyses are likely confounded and should be interpreted with caution. Nevertheless, given biological plausibility and the associations observed in univariable analyses, we hypothesize that histopathologic grade may still retain prognostic relevance in larger populations, although in our dataset, none of the three grading systems remained independently prognostic after multivariable adjustment.

The prognostic value of histopathological grade in cohorts exclusively comprising patients with MIBC has been scarcely explored in the literature [1, 2]. Several research groups and the EAU guidelines assign only limited importance to grade in MIBC [2, 14, 15]. Of note and to the best of our knowledge, large-scale studies directly comparable to our approach using multiple classification systems for grade are absent. Our findings contribute to this discussion by demonstrating that the independent prognostic importance of histopathological grade is limited, as none of the grading schemes showed a significant association with DSS in the multivariable analyses. This underscores the need for further research to refine the role of grading in risk stratification of MIBC.

Efforts have been made to enhance the prognostic utility of histopathological evaluation in MIBC. With respect to histological subtypes of urothelial carcinoma - as opposed to pure urothelial carcinoma - distinct survival outcomes have been demonstrated, depending on the specific subtype [16]. However, dividing patients into individual histological subtypes often results in small subcohorts. In our cohort, when all subtypes were analyzed collectively and compared to pure urothelial carcinoma, no significant differences in DSS were observed (data not shown). To ensure transparency, we also performed multivariable analyses restricted to patients with pure urothelial carcinoma (Supplementary Table 2), which yielded results consistent with those obtained in the full cohort. These findings indicate that the model’s prognostic performance is not substantially influenced by the inclusion of histological subtypes.

Further, Eckstein et al. proposed a novel histological scoring system for MIBC aimed at improving the prediction of disease aggressiveness and patient outcomes [15]. Eckstein et al. highlighted the utility of a multidimensional approach that integrates traditional histopathological parameters such as grade with features of tumor biology like tumor budding as marker of epithelial-mesenchymal transition, features of tumor microenvironment like tumor-infiltrating lymphocytes, and growth/spreading patterns [15].

Besides histopathological features, molecular biomarkers must also be considered in urothelial carcinoma, as, among others, demonstrated by the Sjödahl et al. [17]. They showed that, based on gene expression profiles, prognostic urothelial subtypes could be identified that did not fully align with traditional histopathological stage and grade, suggesting that molecular phenotypes represent intrinsic properties of the tumor [17]. Consequently, combinations of histopathological markers and molecular biomarkers are being investigated for more accurate prediction of outcomes in MIBC [2, 18, 19]. While our study questions the importance of grade as a foundational parameter, it also underscores the potential for enhanced risk stratification through the inclusion of additional tumor characteristics. Future studies are warranted to explore how conventional grading systems can be effectively integrated or replaced with novel approaches to refine prognostic accuracy and improve personalized patient management strategies.

This study has several limitations. Firstly, our patient-population consisted of individuals who underwent upfront RC. Nowadays, neoadjuvant cisplatin-based chemotherapy is recommended for eligible patients [2]. Additionally, neoadjuvant immunotherapies for MIBC are currently under investigation. However, a significant proportion of MIBC patients remains ineligible for neoadjuvant systemic therapy due to frailty, comorbidities and/or impaired renal function [2, 20, 21]. Consequently, the findings of this study may not be fully generalizable to patients treated with neoadjuvant therapies. Further research is warranted to address this limitation. Secondly, pathology review was performed locally rather than centrally, introducing a potential source of bias. Third, the pathology review was conducted prior to 2022, resulting in two patients being classified as LG according to WHO2004, although they exhibited histological subtypes that are now automatically classified as HG under the current edition of the WHO classification. Fourth, the inherent limitations of a retrospective study design, such as potential selection and cohort bias, and the lack of standardized prospective data collection, apply. While this study includes a large multicenter cohort of MIBC patients treated with upfront RC, the relatively small number of lower-grade tumors (LG 2.4%, G1/2 6.6%) limits statistical power, increases uncertainty of the estimates, and warrants cautious interpretation of the prognostic implications of grade in MIBC.

## Conclusion

Based on our findings, we concluded that histopathological grade in MIBC should be interpreted with caution, particularly given that none of the three classification systems for grade demonstrated a significant influence on DSS in our multivariable analyses. Further research with even larger cohorts is warranted to clarify its prognostic role and potential integration with other prognostic parameters like certain histological subtypes and (bio)markers.

## Supplementary Information

Below is the link to the electronic supplementary material.

Supplementary Material 1

## Data Availability

The data that support the findings of this study are available from the corresponding author upon reasonable request. Restrictions apply to the availability of these data due to ethical and privacy considerations.

## Abbreviations

EAU: European Association of Urology
DSS: Disease-specific survival
HG: High-grade
IQR: Interquartile range
LG: Low-grade
LVI: Lympho-vascular invasion
MIBC: Muscle-invasive urothelial carcinoma
NMIBC: Non-muscle-invasive bladder cancer
PUNLMP: Papillary urothelial neoplasm of low malignant potential
RC: Radical cystectomy
WHO: World Health Organization
