# Supplementary material for: Is histological grade a useful parameter in muscle-invasive urothelial bladder cancer? Results from a multicenter study on the impact of different grading systems on disease-free survival after upfront radical cystectomy
Source: World J Urol. 2025 Nov 20;43(1):705. doi: 10.1007/s00345-025-06089-z (PMC12634793; doi:10.1007/s00345-025-06089-z)
Supplement: Supplementary file 1 — Supplementary Material 1 [file 345_2025_6089_MOESM1_ESM.docx]

**Table of Contents**

Supplementary Table S1:…………………………………….…………………………………………………………………2

Variance Inflation Factors for fixed covariates included in the Cox proportional hazards models.

Supplementary Table S2:………………………………………………………………………….……………………………3

Multivariable Cox-Regression analysis of the impact of the WHO1973, WHO2004,
and Hybrid grading scheme, including the base model, on disease-specific survival in patients with pure urothelial carcinoma.

**Supplementary Table S1**:

**Table S1** VIF values for fixed covariates included in the Cox proportional hazards models. VIF values are shown for the base model (all cases) and the model restricted to pure urothelial carcinoma only.

| **Base Model** | **Variable** | **VIF** |
| --- | --- | --- |
| All cases |  |  |
|  | Age (years) | 1.10 |
|  | Stage | 1.05 |
|  | Lympho-vascular invasion | 1.11 |
|  | Positive nodes | 1.20 |
|  | Positive margins | 1.05 |
|  | Adjuvant chemotherapy | 1.20 |
|  | Era of CE | 1.03 |
| Pure urothelial carcinoma only |  |  |
|  | Age (years) | 1.10 |
|  | Stage | 1.04 |
|  | Lympho-vascular invasion | 1.09 |
|  | Positive nodes | 1.20 |
|  | Positive margins | 1.04 |
|  | Adjuvant chemotherapy | 1.22 |
|  | Era of CE | 1.03 |

VIF = Variance Inflation Factor; CE=Cystectomy

**Supplementary Table S2**:

**Table S2** Multivariable Cox-Regression analysis of the impact of the WHO1973, WHO2004, and Hybrid grading scheme, including the base model, on disease-specific survival in patients with pure urothelial carcinoma (n=834).

|  | **base model** |  | **WHO 1973^a^** |  | **WHO 2004** |  | **Hybrid (Ref: LG)** |  |
| --- | --- | --- | --- | --- | --- | --- | --- | --- |
| **Variable** | **HR (95% CI)** | ***p*-value** | **HR (95% CI)** | ***p*-value** | **HR (95% CI)** | ***p*-value** | **HR (95% CI)** | ***p*-value** |
| age (years) | 1.01 (1.00-1.02) | 0.225 | 1.01 (1.00-1.02) | 0.216 | 1.01 (1.00-1.02) | 0.198 | 1.01 (1.00-1.02) | 0.201 |
| stage (Ref: pT2) |  |  |  |  |  |  |  |  |
| pT3 | 1.60 (1.24-2.07) | <.001 | 1.57 (1.21-2.03) | <.001 | 1.58 (1.23-2.04) | <.001 | 1.57 (1.22-2.03) | <.001 |
| pT4 | 2.00 (1.48-2.72) | <.001 | 1.97 (1.45-2.67) | <.001 | 1.99 (1.47-2.70) | <.001 | 1.98 (1.46-2.69) | <.001 |
| LVI | 1.38 (1.11-1.71) | 0.003 | 1.38 (1.11-1.71) | 0.003 | 1.37 (1.10-1.69) | 0.004 | 1.37 (1.11-1.70) | 0.004 |
| positive nodes | 2.04 (1.62-2.57) | <.001 | 2.02 (1.60-2.54) | <.001 | 2.02 (1.61-2.55) | <.001 | 2.02 (1.60-2.54) | <.001 |
| positive margins | 1.48 (1.10-1.98) | 0.01 | 1.47 (1.10-1.98) | 0.01 | 1.48 (1.10-1.99) | 0.01 | 1.48 (1.10-1.99) | 0.010 |
| adj. chemotherapy | 0.94 (0.74-1.20) | 0.641 | 0.95 (0.74-1.21) | 0.658 | 0.95 (0.74-1.21) | 0.662 | 0.95 (0.74-1.21) | 0.664 |
| era of CE (Ref: ≤ 2000) |  |  |  |  |  |  |  |  |
| 2001-2010 | 1.05 (0.80-1.37) | 0.728 | 1.04 (0.80-1.35) | 0.791 | 1.05 (0.80-1.37) | 0.745 | 1.04 (0.80-1.36) | 0.768 |
| 2011-2020 | 0.97 (0.64-1.48) | 0.898 | 0.98 (0.64-1.48) | 0.907 | 0.98 (0.64-1.49) | 0.911 | 0.98 (0.64-1.49) | 0.912 |
| Treatment center^b^ | -^c^ |  | -^d^ |  | -^e^ |  | -^f^ |  |
| **+ Grading** | **-** | **-** | **1.35 (0.84-2.17)** | **0.210** | **1.82 (0.81-4.12)** | **0.150** | HG/G2  **1.62 (0.61-4.32)** | **0.332** |
|  |  |  |  |  |  |  | HG/G3  **1.83 (0.81-4.14)** | **0.147** |
| ^a^ dichotomized into G1/2 and G3. ^b^ treatment center was included as a frailty (random effect). ^c^variance of the term = 0.0329, p-value from likelihood-ratio test = <.001. ^d^variance of the term = 0.0303, p-value from likelihood-ratio test = <.001. ^e^variance of the term = 0.0356, p-value from likelihood-ratio test = <.001. ^f^variance of the term = 0.0337, p-value from likelihood-ratio test = <.001.  Adj. = adjuvant; CE = cystectomy; CI = confidence interval; HG = high grade; HR = hazard ratio; LG = low grade; LVI = Lympho-vascular invasion; Ref = reference category. | | | | | | | | |
